# Infective Endocarditis Observed With Cryoglobulinemic Vasculitis

**DOI:** 10.7759/cureus.10620

**Published:** 2020-09-23

**Authors:** Kevin Liu, Andrew Newman

**Affiliations:** 1 Dermatology, Midwestern University, Arizona College of Osteopathic Medicine, Glendale, USA; 2 Dermatology, Affiliated Dermatology, Scottsdale, USA

**Keywords:** cryoglobulin, vasculitis, infective endocarditis

## Abstract

Cryoglobulinemic vasculitis is most commonly observed in the setting of hepatitis C infection. However, many other etiologies have been identified as well. We herein report a case of cryoglobulinemic vasculitis in a 56-year-old male secondary to infective endocarditis. The patient presented with a five-week history of a painful, purpuric rash and was found to have positive blood cultures, elevated cryoglobulins, and vasculitis on histology. He recovered after treatment with intravenous antibiotics and steroids. Although rarely identified as a cause of cryoglobulinemic vasculitis, infective endocarditis must be considered on the differential diagnosis as a delay in treatment can worsen the prognosis for the patient.

## Introduction

Cryoglobulins are circulating immunoglobulins that can precipitate in cold environments and cause vasculitis, particularly affecting the skin, kidneys, and peripheral nerves. Although associated with hepatitis C infection in up to 90% of cases, cryoglobulinemia has been linked to many other infections, such as the hepatitis B virus and HIV, and autoimmune conditions like Sjogren’s syndrome, systemic lupus erythematosus, and rheumatoid arthritis [1]. Since most cases of cryoglobulinemia have an underlying cause, definitive treatment typically attempts to correct the underlying disorder once it is identified. Therefore in patients found to have cryoglobulinemic vasculitis, accurate identification of the cause of the disorder is crucial to developing a treatment plan. We present a case of cryoglobulinemic vasculitis observed with infective endocarditis (IE).

## Case presentation

**Figure 1:**
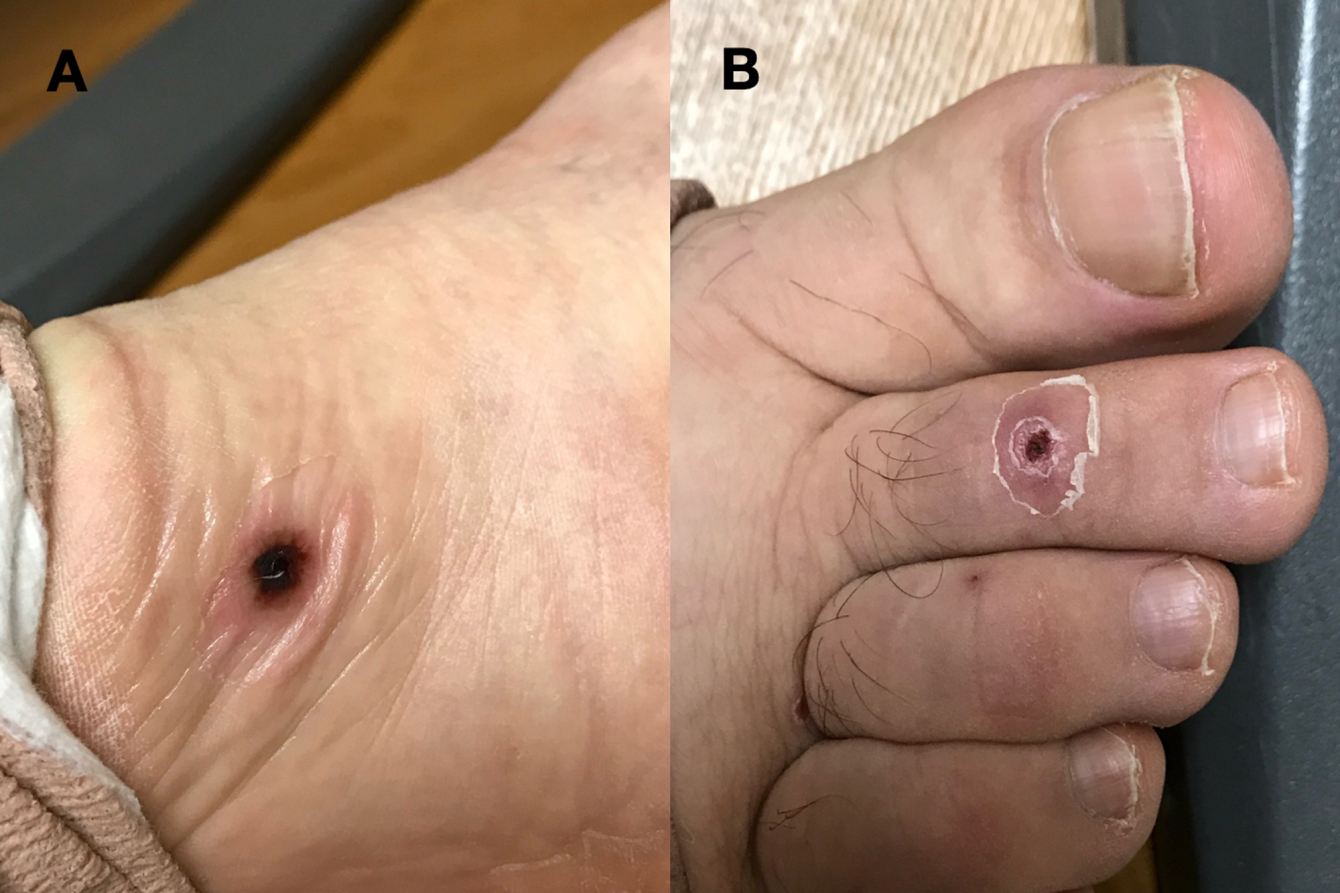
Purpuric lesion on the right foot (A) and ulcer on dorsal right second toe (B)

**Figure 2:**
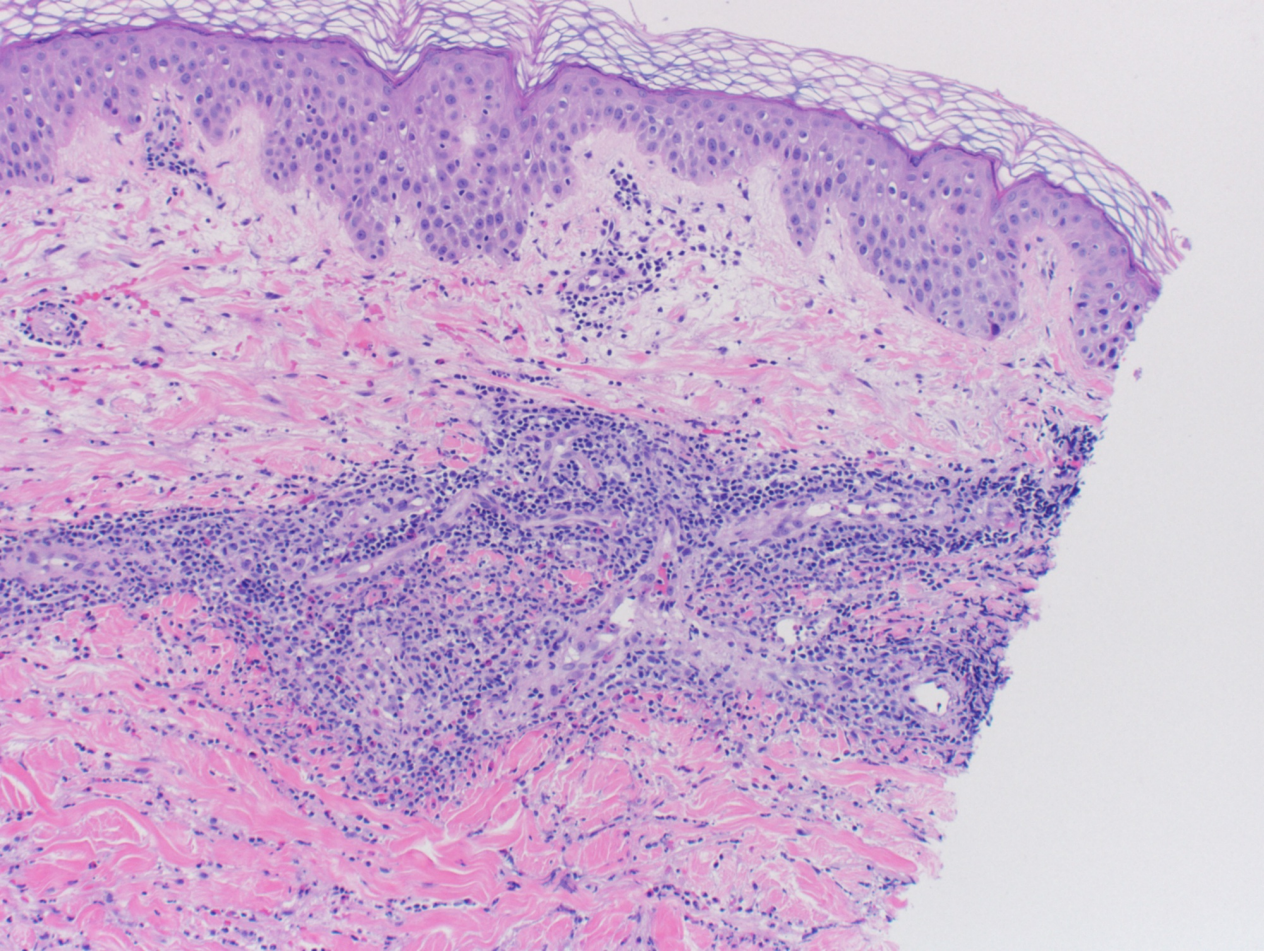
Histological staining with hematoxylin and eosin reveals leukocytoclasia and mild lymphocytic infiltrate of the small capillaries (10x magnification)

A 56-year-old male with no prior medical history except for chronic low back pain was admitted to the hospital for a painful rash over his legs, feet, and hands with associated malaise for five weeks. The patient stated his rash became more prominent when he was cold. The patient denied any history of drug use. He denied any fever or arthralgias. A punch biopsy performed by a dermatologist three weeks prior revealed only mild spongiosis with a superficial perivascular lymphocytic infiltrate. The dermatology service was consulted for the rash. His physical examination included a toxic-appearing man breathing room air with subtle palpable purpura and scattered 1-3 mm ulcers mainly on the acral surfaces of his hands and feet but also bilateral lower legs (Figure 1). The patient was suspected to have leukocytoclastic vasculitis which was subsequently confirmed by histological examination. His labs were pertinent for methicillin-sensitive Staphylococcus aureus positive blood cultures x2, significantly elevated immunoglobulin G (IgG) and immunoglobulin M (IgM) cryoglobulins, and one small vegetation on the aortic valve found on transthoracic echocardiogram. Urinalysis showed trace proteinuria. Erythrocyte sedimentation rate (ESR) was 92 mm/hr, C-reactive protein (CRP) was 44 mg/L, complete blood count (CBC), and liver function tests (LFTs) were unremarkable. Hepatitis panel, HIV, Coccidioidomycoses titers, anti-nuclear antibody, and anti-neutrophil cytoplasmic antibodies were negative, and protein electrophoresis was within normal limits. Creatinine was 1.6 mg/dL, elevated from a baseline of 0.9 mg/dL, and glomerular filtration rate (GFR) was 56 mL/min. Urine output was adequate. Extensive coagulopathy studies found no abnormalities. Venous and arterial Doppler ultrasound studies performed in bilateral lower extremities had no significant findings. Two punch biopsies of separate lesions showed vasculitis in small capillaries (Figure 2). No septic or thrombotic emboli were appreciated. The patient was diagnosed with cryoglobulinemic vasculitis. He was started on intravenous (IV) cefazolin, but the cutaneous lesions persisted for weeks after beginning antibiotic therapy. After coordination with infectious disease and dermatology services, the patient was started on 60 mg of IV methylprednisolone every six hours for four days. The patient reported significant improvement regarding his rash and overall well-being after two days of initiating corticosteroid therapy. He was given a two-week course of prednisone, tapering from 40 mg to 5 mg, topical triamcinolone 0.1% ointment, and was instructed to continue cefazolin for a total course of eight weeks.

## Discussion

Cryoglobulinemia entails the presence of abnormal antibodies that are water-soluble in warm environments yet precipitate in colder conditions. Immune complexes produced from precipitation cause inflammation that affects the small vessels, most notably in the skin and joints [2]. The most typical symptoms are fatigue, purpura, and arthralgia. Renal and nervous system involvement is also commonly seen, presenting as proteinuria, microscopic hematuria, and mononeuritis multiplex. Diagnosis is made based on clinical, laboratory, and histological evidence, and typically relies on the identification of cryoglobulins in the serum [1]. Type III cryoglobulinemia, involving polyclonal IgG and IgM, results from hepatitis C infection in the majority of cases. However, it can also be caused by autoimmune disorders, lymphoproliferative disease, and other chronic infectious states. Treatment of type III cryoglobulinemic vasculitis usually involves a combination of immunosuppressive agents, such as corticosteroids, and therapy directed at the underlying cause. In the case of infective endocarditis, appropriate therapy would be an antibiotic course based on blood culture sensitivities.

Although classically associated with hepatitis C, the presence of cryoglobulins in patients with IE has previously been reported [3-4]. In these patients, the mixed, type III cryoglobulinemia is most often seen, with IgG, IgM, and immunoglobulin A (IgA) observed in the cryoprecipitates. These circulating immune complexes typically are not significant enough to result in appreciable systemic manifestations. Additionally, staphylococcal IE is often rapidly progressive, so the onset of symptoms, diagnosis, and treatment typically occurs before the development of quantities of cryoglobulins required to elicit symptoms. More commonly seen are vascular lesions resulting from septic emboli, such as splenic and renal infarcts, Janeway lesions, and splinter hemorrhages. Because of its high mortality rate, prompt diagnosis and treatment are crucial in the management of IE [5]. Treating a patient with IE with corticosteroids may impair the patient’s ability to fight the infection, leading to an exacerbation of their condition. It is, therefore, essential to consider IE when evaluating a patient with cryoglobulinemia.

Despite not being commonly observed in this setting, cryoglobulinemic vasculitis has been documented as the presenting symptom of IE [6-7]. In a case published in 2017, a patient presented with a non-blanching purpuric rash on her lower extremities, anemia, and blood and trace protein on urine dipstick [8]. Another case was described in 2006 in which a patient with cryoglobulinemic vasculitis secondary to Coxiella burnetii endocarditis [9]. This patient also presented with lower extremity purpura and renal disease, and additionally had normal blood cultures and no vegetations or valvular dysfunction was seen on transesophageal echocardiograph. Despite the lack of classical IE findings, a diagnosis of Q fever endocarditis was made, and antibiotic therapy was started promptly. However, in a similar case published in the Journal of The American Society of Nephrology, a patient with lower extremity vasculitis, impaired renal function, negative blood cultures, and no vegetations on echocardiogram was initially diagnosed with essential cryoglobulinemia [10]. When he was started on prednisone, his condition worsened after briefly improving. His symptoms resolved after the steroids were stopped, and antibiotics were begun.

As with the cases above, our patient presented with a purpuric rash on his legs and mild renal impairment. Unlike the previous cases, however, his skin lesions were also present on the upper extremity. He had growth of methicillin-sensitive Staphylococcus aureus on blood cultures and vegetation on the aortic valve seen on transthoracic echocardiogram, strengthening the case for IE as a diagnosis. The patient’s IE exhibited a relatively indolent course of illness. We believe that this slow progression uniquely allowed for the development of cryoglobulinemia significant enough to be a prominent contributor to his overall IE disease course, rather than showcasing the more classical IE features: septic emboli, internal organ infarcts, and other vascular phenomena. In his case, the patient was administered antibiotics in conjunction with systemic and topical corticosteroids, which addressed both the infectious and inflammatory nature of his condition.

## Conclusions

Infective endocarditis may, on rare occasions, cause cryoglobulinemia, which typically features a purpuric lower extremity rash and renal dysfunction. Clinicians should appreciate the high mortality rate of IE, especially if not rapidly detected. A treatment regimen consisting of corticosteroids without antibiotics may worsen patients with IE due their immunosuppressive effects. Therefore, failure to consider IE in the differential diagnosis of cryoglobulinemia may seriously compromise a patient’s prognosis.
